# Limitations of Muscle Ultrasound Shear Wave Elastography for Clinical Routine—Positioning and Muscle Selection

**DOI:** 10.3390/s21248490

**Published:** 2021-12-20

**Authors:** Alyssa Romano, Deborah Staber, Alexander Grimm, Cornelius Kronlage, Justus Marquetand

**Affiliations:** 1 Department of Epileptology, Hertie-Institute for Clinical Brain Research, University of Tübingen, 72074 Tübingen, Germany; alyssa.romano@student.uni-tuebingen.de (A.R.); deborahstaber@hotmail.com (D.S.); alexander.grimm@med.uni-tuebingen.de (A.G.); cornelius.kronlage@med.uni-tuebingen.de (C.K.); 2 Department of Neural Dynamics and Magnetoencephalography, Hertie-Institute for Clinical Brain Research, University of Tübingen, 72074 Tübingen, Germany; 3 MEG-Center, University of Tübingen, 72074 Tübingen, Germany

**Keywords:** SWE, elastography, elasticity, optimized, rigid SWE-protocol

## Abstract

Shear wave elastography (SWE) is a clinical ultrasound imaging modality that enables non-invasive estimation of tissue elasticity. However, various methodological factors—such as vendor-specific implementations of SWE, mechanical anisotropy of tissue, varying anatomical position of muscle and changes in elasticity due to passive muscle stretch—can confound muscle SWE measurements and increase their variability. A measurement protocol with a low variability of reference measurements in healthy subjects is desirable to facilitate diagnostic conclusions on an individual-patient level. Here, we present data from 52 healthy volunteers in the areas of: (1) Characterizing different limb and truncal muscles in terms of inter-subject variability of SWE measurements. Superficial muscles with little pennation, such as biceps brachii, exhibit the lowest variability whereas paravertebral muscles show the highest. (2) Comparing two protocols with different limb positioning in a trade-off between examination convenience and SWE measurement variability. Repositioning to achieve low passive extension of each muscle results in the lowest SWE variability. (3) Providing SWE shear wave velocity (SWV) reference values for a specific ultrasound machine/transducer setup (Canon Aplio i800, 18 MHz probe) for a number of muscles and two positioning protocols. We argue that methodological issues limit the current clinical applicability of muscle SWE.

## 1. Introduction

Shear wave elastography (SWE) is a specialized modality in ultrasound imaging that allows the measurement of elasticity, or stiffness, of various bodily tissues. Shear waves propagate perpendicularly to longitudinal ultrasound waves generated by an ultrasound transducer [1]. Elasticity can then be estimated from shear wave velocity (SWV), assuming a linear elastic, isotropic and homogenous tissue model, using the equation for shear elastic modulus (µ), where µ = ρV_s_^2^ [2]. In this equation, ρ represents the density of the examined tissue (ρ for muscle can be estimated to be 1000 kg/m^3^) and V_s_ denotes the SWV. Thus, an increase in shear elastic modulus, or stiffness, is positively correlated with an increase in SWV [3].

In clinical medicine, SWE has—most notably—been researched and established for the staging of liver fibrosis (Ferraioli et al., 2015) and the diagnosis and classification of tumors in breast, thyroid and prostate tissue [1,4,5,6]. Muscle tissue can also be analyzed with SWE so that neuromuscular medicine has a potential new modality for clinical application. For example, group-level differences in muscle SWE for at least some muscles in comparison to healthy controls have been demonstrated for Duchenne muscular dystrophy [7,8] as well as inflammatory myopathies [9]; a correlation of muscle elasticity as determined by SWE with muscle strength has been described in inclusion-body myositis [10]; muscle SWE has been shown to resolve delayed muscle relaxation in myotonic muscle disorders [11].

In addition to general technical factors such as vendor-specific implementations of SWE, probe load and measurement depth, muscle tissue poses specific challenges for SWE. For example, the natural mechanical anisotropy of muscle, its varying architecture in terms of pennation, and changes in shear wave speed that are independent of elasticity and are caused by nonzero tensile stress resulting from passive stretch and/or muscle contraction, as well as changes in the elastic modulus, itself, caused by passive stretch and contraction, can all contribute to variability and confound measurements [12,13,14,15,16,17,18].

In clinical diagnostics, conclusions usually need to be made on an individual patient level cross-sectionally, i.e., measurements of a single patient are compared to established reference values. In this respect, not only good measurement reliability but also conditions with low overall variability in the reference measurements of healthy subjects are desirable. Additionally, examination convenience (i.e., feasible positioning also in patients with disabilities) and time-efficiency are critical for the applicability of muscle SWE in routine clinical diagnostics.

So far, no general reference values for muscle SWE have been established [19]. Lacourpaille et al. [20] described a measurement protocol with different subject positions aiming for low muscle extension and reported good intra- and inter-observer reliability of ultrasound SWE in nine muscles. Dubois et al. [21] examined intra- and inter-operator reliability of ultrasound SWE in 11 lower-extremity muscles and found that, while measurements in a stretched position had a tendency to be less reliable, the difference was not statistically significant, leading to the interpretation that there was no effect. They also report lower reliability for deeper muscles (soleus, biceps femoris).

In this work, we aim to provide a clinically feasible protocol for muscle ultrasound SWE by (1) characterizing different limb and truncal muscles in terms of inter-subject variability of SWE measurements, (2) comparing two protocols with different limb positioning in a trade-off between examination convenience and SWE measurement variability and (3) providing SWE SWV reference values for a specific ultrasound machine/transducer setup (Canon Aplio i800, 18 MHz probe, Canon Medical Systems Europe B.V., Zoetermeer, The Netherlands) for a number of muscles and the two subject positioning protocols.

## 2. Materials and Methods

### 2.1. Participants

All 52 healthy volunteers recruited had no history or clinical signs of neuromuscular disease. In general, the majority of the participants maintained an active physical lifestyle, which might be represented by the average normal to low-normal BMIs (Table 1). All participants were asked to refrain from doing any athletic, strenuous activity 48 h before the examination to ensure the muscles were in an optimal, relaxed state. Only muscles on the right side of the body were investigated. All participants were over the age of 18 and provided informed consent through written documentation. The ethics committee of the University of Tübingen approved the study (project number 641/2020BO2) and the examinations were carried out abiding by the Declaration of Helsinki.

This study was carried out using 2 Protocols: In Protocol 1 (clinical feasibility), subjects were examined in a similar fashion as routine clinical, where B-mode muscle ultrasound is usually performed—that is, in a supine and, in turn, prone position, without strict requirements regarding limb positioning. In this protocol, the least possible adjustments were made during examination to optimize time efficiency and patient comfort. In contrast, Protocol 2 (optimized, rigid SWE-protocol) was centered around achieving a reproducible, relaxed positioning of each examined muscle. This protocol is more demanding for subjects (potentially patients with significant disabilities) and requires significantly more time. Different participants were assessed in each protocol in order to collect more data representative of the general population. The details of the positions are shown in Table 2, which also provides a comparison of muscle positioning in other SWE studies published to date.

In both protocols, young healthy subjects were investigated (25 participants for Protocol 1 and 27 participants in Protocol 2). Further descriptions of these two groups are found in Table 1.

### 2.2. SWE Measurements and Subject Positioning

**Figure A1:**
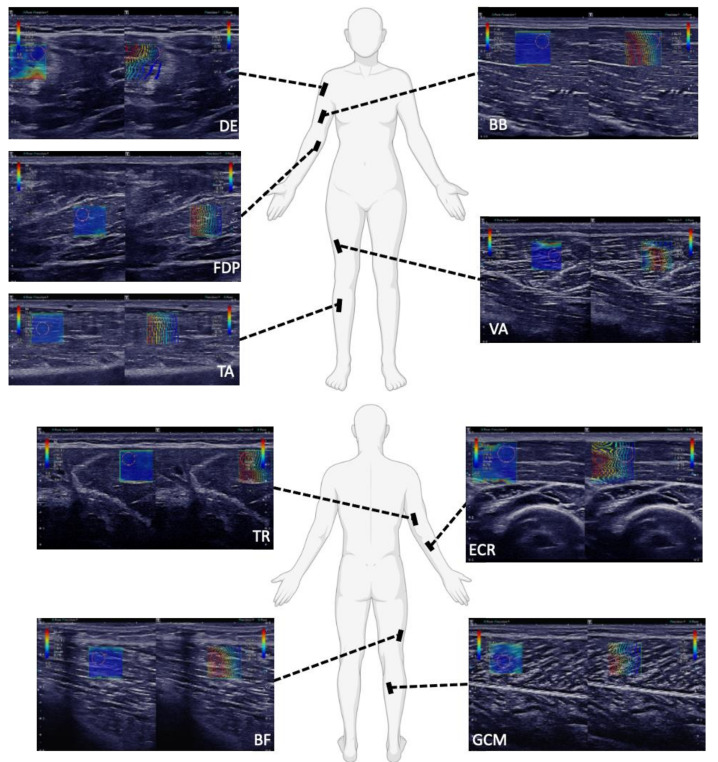
SWE images of the examination of a 32-year-old male in Protocol 2 (optimized, rigid SWE-protocol): SWE pictures of the deltoid (DE), flexor digitorum profundus (FDP), tibialis anterior (TA), biceps brachii (BB) and vastus lateralis (VA) muscle, triceps brachii (TR), biceps femoris caput longum (BF), extensor carpi radialis (ECR) and gastrocnemius caput mediale (GCM) muscle are shown. On the left side of each picture, the B-mode images in gray scale are overlaid with SWV data in color. The cooler colors, such as the blue in these pictures, depict slower shear wave speeds, typically ranging from 0–6 m/s. As predicted, in Protocol 2 (optimized, rigid SWE-protocol), the muscles were positioned in optimally relaxed states, to avoid strain, which was demonstrated by the consistent blue coloring within the SWE pictures. On the right side of each picture, the shape of the shear waves is displayed with lines. The blue lines represent the origin of the shear waves and the red lines represent the change in the shear waves as they propagate accordingly through the muscle. In these pictures, the greater depth of acquisition required for FDP and DE can be seen. The layer of subcutaneous fat above the DE was typically thicker than for TA, BB and VA. Additionally, the greater pennation angle of DE is illustrated in that the muscle fibers could not be completely optimally displayed to parallel in the longitudinal plane. Alternatively, the path of the muscle fibers of FDP, TA, BB and VA could be displayed well in the longitudinal plane.

For optimal measurement conditions for each muscle, SWE-recordings were completed within 20–30 s. Since the utilized ultrasound device Canon Aplio i800 records one sample per second, 20–30 images/samples were available for estimating SWE. Of these 20–30 images, three random images were chosen and a circular region of interest (ROI) was placed in a homogeneous region of the muscle for SWV measurements (Figure A1). The three SWV measures were averaged, resulting in a single value used in subsequent analyses.

In Protocol 1 (clinical feasibility), for the muscles of the upper extremity, volunteers were asked to lay on their back, in the supine position, with their arms stretched out at their side, resting on the examination table, palms facing up. When measuring the SWE of the back muscles, participants were asked to lay on the examination table in the prone position, with their hands at their side. For the lower extremity muscles, volunteers were asked to lay on their stomach or back depending on if the muscle was on the ventral or dorsal side of the body.

In Protocol 2 (optimized, rigid SWE-protocol), each muscle was examined in a position in which the muscles were not strained in terms of stretch or contraction, but in the most optimally relaxed state. Since positioning could not be optimized for the paravertebral back muscles, these were not included again (see also Results). A detailed description of the joint positions used for each muscle, as well as a comparison of our protocol to other studies can be found in Table 1.

### 2.3. Statistical Analysis

The statistical analysis was performed using SPSS 27.0 Software (IBM, Armonk, New York, NY, USA). For each protocol, descriptive statistics were computed—including the mean, median, range, standard deviation and variance. Normal distribution was assessed using the Shapiro–Wilk Test, and as data was not normally distributed, only tests for non-normally distributed data were used. The SWV values, variance and biometric parameters between Protocol 1 (clinical feasibility) and Protocol 2 (optimized, rigid SWE-protocol) were compared using the Mann–Whitney U Test. For all tests, the significance level was set to *p* < 0.05. All graphs were created using SPSS, JMP (SAS, Cary, NC, USA) and Microsoft Excel.

## 3. Results

**Figure 1:**
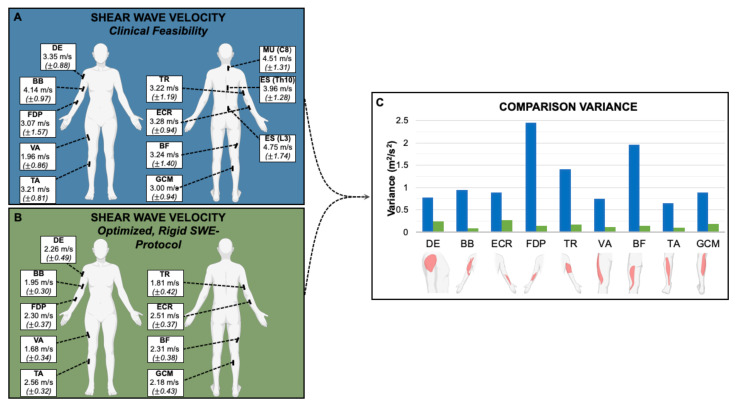
Representation of the muscle measurement locations with the average SWV in m/s and the corresponding standard deviation (±) of Protocol 1 (**A**) and 2 (**B**) and comparison of the variance between them (**C**): The variances of measurements acquired using Protocol 2 (optimized, rigid SWE-protocol) were significantly lower (*p* < 0.001) than under Protocol 1 (clinical feasibility). DE = deltoideus. BB = biceps brachii. ECR = extensor carpi radialis. FDP = flexor digitorum profundus. TR = triceps brachii. MU (C8) = multifidus (C8). ES (Th10) = erector spinae (Th10). ES (L3) = erector spinae (L3). VA = vastus lateralis. BF = biceps femoris (caput longum). TA = tibialis anterior. GCM = gastrocnemius (caput mediale).

The average SWV for all muscles except vastus lateralis (VA) were significantly higher (*p* < 0.05) when measured according to Protocol 1 (clinical feasibility) than in Protocol 2 (optimized, rigid SWE-protocol) (Table 3, Figure 1).

Additionally, a comparison of the average SWV to previously published SWV findings can be found in Table 4. Differences between studies often do not exceed variability in the respective groups. Besides subjects’ positioning, technical factors such as the used equipment affect the measurements.

When focusing on examination time efficiency (Protocol 1), the variances of measurements were significantly higher (*p* < 0.001) than when controlling specifically for joint position (Protocol 2). The autochthonous back muscles—MU (C8), ES (Th10) and ES (L3)—exhibited high variance in Protocol 1 (clinical feasibility). These muscles were small, lay deep and were particularly difficult to examine—since positioning could not be optimized, they were not further investigated in Protocol 2 (optimized, rigid SWE-protocol).

## 4. Discussion

Ultrasound SWE has been established for many clinical applications, such as the assessment of liver, breast or thyroid lesions. However, whereas ‘conventional’ B-mode ultrasound is being widely clinically used complementary to electrodiagnostic studies [23], the adoption of muscle SWE in the field of neuromuscular medicine is still met by specific methodological challenges. The presented results demonstrate clinical SWE examination protocols in terms of practical feasibility, muscle selection and patient positioning.

In group-level comparisons, muscle elasticity as determined by ultrasound SWE has been found to be altered in different myopathies [7,8,10], e.g., elasticity is likely reduced in idiopathic inflammatory myopathies (IIM) when compared to healthy controls [24,25]. Such features may serve as diagnostic indicators. The magnitude of effect, however, is relatively small compared to the variability of measurements, which limits the diagnostic performance of muscle ultrasound SWE for classifying individual patients cross-sectionally.

In this work, we study the inter-subject variability of muscle SWE measurements in healthy controls. Low variability facilitates the detection of pathological changes in groups with small sample sizes and especially in individual subjects, thus it represents a prerequisite and an indicator for clinical utility of SWE measurements.

When comparing all muscles examined in this study, the biceps brachii and tibialis anterior muscles exhibit the lowest variability. We attribute this to their superficial location, sufficiently large size and low degree of pennation. In contrast, in autochthonous back muscles (multifidus, erector spinae), we observed high SWV values and high variability, which may be caused by greater measurement depths, smaller dimensions and more complex architectures of the respective muscles as well as tonic, involuntary contractions. It should be noted, however, that methodological requirements for the selection of muscles to be examined by muscle ultrasound SWE also need to be weighed against clinical aspects in diagnostic applications. For example, myopathies are not necessarily diffuse, but may exhibit different and typical patterns of involvement. Therefore, it may not be suitable to examine only technically favorable sites in such cases.

Besides muscle selection, we also find that reproducible and precise positioning is key in order to obtain muscle SWE data with low inter-subject variability. When subjects were simply lying supine or prone (Protocol 1, clinical feasibility), mimicking routine clinical (B-mode) muscle ultrasound examinations [23], we observed a high variability of measurements (Figure 1). The more detailed and rigorous examination, Protocol 2, results in significantly lower variability. On the downside, it requires more time and is more demanding for examined subjects, which is not of concern for healthy volunteers, but may pose a challenge for neurological patients with significant disabilities. Subject positioning, to obtain less passive extension of the examined muscles, predictably resulted in lower absolute SWV results. It is well known that muscle tensile force, as induced by passive extension, correlates with muscle elasticity determined by ultrasound SWE [12,26,27,28,29].

Although the mean age of participants examined according to Protocol 1 in this study was significantly higher than in the Protocol 2 group, we did not research further the possible effect of age in our cohorts, considering all participants were relatively young (mean age 26.5–33 years). In this respect, there is conflicting evidence as to whether SWE increases or decreases with age [9,30], however, investigating the effect of age on muscle SWE was not the aim of our study.

Similar to Protocol 2 (optimized, rigid SWE-protocol), Lacourpaille et al. (2012) [16] reported good intra- and inter-observer reliability, where subject positioning aimed to achieve low muscle extension. Dubois et al. (2015) [17] studied both stretched and relaxed positions, resulting in a statistically non-significant trend towards less reliable measurements in the stretched conditions. Sarabon et al. [31] conducted a reliability study and found lower minimal detectable changes of biceps femoris elasticity when relaxed compared to stretched. These findings, along with ours in this study, support the argument that SWE is most reliable when the muscles are not passively stretched, since the SWV in these relaxed positions consistently demonstrate in lower variability.

When comparing muscle SWE in patients with muscle disease to healthy controls, Alfuraih et al. (2018) searched for the distinction of idiopathic inflammatory myopathy (IIM) patients to controls. In this study, reported areas under the receiver operating characteristic curve (AUROC) ranged from 0.51 up to 0.93, depending on the examined muscle and the position. AUROCs were not significantly different from 0.5 for measurements in the different parts of the quadriceps femoris muscle when passively stretched, but exhibited good performance of up to 0.87 when in a resting state. Comparing patients with Duchenne muscular dystrophy (DMD) to healthy controls, Lacourpaille et al. (2015) [7] described an increased stiffness in DMD irrespective of passive stretching in the gastrocnemius, vastus lateralis and triceps brachii muscle. However, in tibialis anterior and biceps brachii, there were differences between groups only in the passively stretched, but not in the relaxed conditions, even though the variability of measurements (standard deviations) were much higher upon stretching, similar to our results. Likewise, Gao et al. (2018) [32] found a difference between patients with chronic post-stroke spasticity and controls in biceps brachii SWV only when the elbow was extended, but not with 90° flexion. In summary, even though examination with minimal passive extension results appears to result in the highest reliability and lowest variability of muscle SWE measurements, only examination in stretched conditions may provide clinically relevant information in some diseases.

Various technical factors need to be considered in SWE. Manufacturers of clinical ultrasound systems use different implementations of the method. Additionally, measurements made even with different transducers on the same device are not directly comparable as parameters, such as the shear wave frequency, differ [13,33]. In principle, reference values need to be established specifically for every technical setup. Here, we provide data for the Canon Aplio i800 with an 18 MHz broadband probe. The comparison of our measurements (Protocol 2—optimized, rigid SWE-protocol) to the literature (Table 4) shows that differences in SWV between studies often do not exceed variability in the respective groups. Still, in order to detect small effects and thereby optimize diagnostic performance, examination modalities and the used equipment needs to be taken into account.

Strengths of this study include the number of muscles that were assessed by SWE, examinations having been performed by a single examiner, and the close resemblance of Protocol 1 (clinical feasibility) to routine clinical practice in ‘conventional’ (B-mode) muscle ultrasound.

The study is limited by the small sample size, the demographics of participants with possibly limited validity for older patient cohorts and the lack of a (re-test) reliability assessment.

In conclusion, our results characterize SWE of various limb and truncal muscles, and highlight that patient positioning, among other technical factors, need to be taken into consideration when performing clinical muscle ultrasound SWE. Furthermore, we provide SWE data for the transducer setup using the Canon Aplio i800 System and 18 MHz probe. Still, the limited number of well-accessible muscles, as well as issues related to time-efficiency and examination feasibility reduce the utility of SWE as a routine diagnostic modality in neuromuscular medicine in comparison to ‘conventional’ muscle ultrasound.

## Figures and Tables

**Table 1:** Summary of participants’ characteristics examined in this study. The two groups did not differ significantly in terms of height, weight or BMI.

Summary of Participants’ Characteristics					
Protocol	Age in Years Mean (SD)	Height in cm Mean (SD)	Weight in kg Mean (SD)	BMI in kg/m^2^ Mean (SD)	Number of Participants Male, Female
**Protocol 1** *(Clinical Feasibility*)	33.0 (13.3)	177.0 (8.8)	72.7 (11.7)	23.1 (2.5)	17, 8
**Protocol 2** (*Optimized, Rigid SWE-Protocol*)	26.5 (3.1)	176.0 (7.6)	67.4 (10.1)	21.7 (2.2)	15, 12
*p*	0.032 *	*0.640*	*0.113*	*0.058*	*0.361*

* There was a significant difference between the two groups in terms of age. *p* is given for comparisons between groups (Mann–Whitney U Test).

**Table 2:** Summary of different positionings for muscle ultrasound SWE, sorted by muscle and by author.

**Joint Position**								
Muscle	This Study (Protocol 2)	Alfuraih et al. (2018 & 2019)	Ewertsen et al. (2016)	Carpenter et al. (2015)	Akagi et al. (2015)	Cortez et al. (2017)	Lacourpaille et al. (2012)	Dubois et al. (2015)
**DE**	Supine, elbow resting on a pillow, arm bent at the elbow 90°							
**BB**	Supine, elbow resting on a pillow, arm bent at the elbow 90°	Supine, elbow resting on a pillow, arm bent at the elbow 90°	Sitting, forearm resting, supinated underarm				Arm bent at the elbow 90°	Prone, 90° bent between legs and thighs
**ECR**	Supine, elbow resting on a pillow, arm bent at the elbow 90°							
**FDP**	Supine, arm stretched out							
**TR**	Left lateral recumbent						Arm at full extension	
**VA**	Supine, legs almost completely stretched out with a small pillow under the knees	Supine, knees fully extended and feet slightly everted		Prone, lower extremity fully supported			Knee fully extended	Sitting upright, hip bent at 90°
**BF**	Sitting, feet flat on the floor	Prone, bent knees (90°), legs rested against a wall						
**TA**	Sitting, lower leg free hanging					Supine, leg extended and heel on the examination table	Knee fully extended, ankle in neutral position	
**GCM** **or** **GCL**	Sitting, lower leg free hanging		Prone, feet relaxed, hanging from bed	Prone, lower extremity fully supported	Prone, hip and knee at ~ 0°, ankle at 20° plantar flexion	Supine, knee flexed and hip in external rotation	Knee bent at 90°, ankle in neutral position	Prone, 90° bent between legs and thighs

DE = deltoideus. BB = biceps brachii. ECR = extensor carpi radialis. FDP = flexor digitorum profundus. TR = triceps brachii. VA = vastus lateralis. BF = biceps femoris (caput longum). TA = tibialis anterior. GCM = gastrocnemius (caput mediale).

**Table 3:** Summary of the average SWE in m/s and standard deviation (SD) for each muscle examined in all protocols and groups of this study.

Muscle	Protocol 1 *(Clinical Feasibility)* *n* = 25	Protocol 2 *(Optimized, Rigid SWE-Protocol)* *n* = 27	*p*
	SWE m/s (SD)	SWE m/s (SD)	
**DE**	3.35 (0.88)	2.26 (0.49)	*<0.001*
**BB**	4.14 (0.97)	1.95 (0.30)	*<0.001*
**ECR**	3.28 (0.94)	2.51 (0.37)	*<0.001*
**FDP**	3.07 (1.57)	2.30 (0.37)	*0.001*
**TR**	3.22 (1.19)	1.81 (0.42)	*<0.001*
**MU (C8)**	4.51 (1.31)		
**ES (Th10)**	3.96 (1.28)		
**ES (L3)**	4.75 (1.74)		
**VA**	1.96 (0.86)	1.68 (0.34)	*0.098*
**BF**	3.24 (1.40)	2.31 (0.38)	*<0.001*
**TA**	3.21 (0.81)	2.56 (0.32)	*<0.001*
**GCM**	3.00 (0.94)	2.18 (0.43)	*<0.001*
**Average**	3.74 (1.15)	2.17 (0.38)	*0.001*

There was a significant difference in the average SWE when comparing Protocol 1 (clinical feasibility) to Protocol 2 (optimized, rigid SWE protocol) in all muscles except VA, as dictated by *p* < 0.05 using the Mann–Whitney U Test. DE = deltoideus. BB = biceps brachii. ECR = extensor carpi radialis. FDP = flexor digitorum profundus. TR = triceps brachii. MU (C8) = multifidus (C8). ES (Th10) = erector spinae (Th10). ES (L3) = erector spinae (L3). VA = vastus lateralis. BF = biceps femoris (caput longum). TA = tibialis anterior. GCM = gastrocnemius (caput mediale).

**Table 4:** Summary of muscle SWE reference values (SWV in m/s), sorted by muscle and by author.

Author	This Study (Protocol 2)	Alfuraih et al. 2018	Alfuraih et al. 2019	Ewertsen et al. 2016 ^	Carpenter et al. 2015 *	Akagi et al. 2015 ^#^	Cortez et al. 2017
**Cohort**	*n* = 27	*n* = 20	*n* = 26	*n* = 10	*n* = 5	*n* = 31	*n* = 16
**Age in years** mean (SD)	26.5 (3.1)	36.7 (11.8)	28.1 (4.1)	Median: 32.5	Range: 27–33	22 (1)	25 (12)
**BMI in kg/m^2^** mean (SD)	21.7 (2.2)	23.0 (3.1)	24.5 (5.3)	All < 31	Not given	21.5	23.2 (2.97)
**Device and Probe**	Canon Aplio i800, PLI 1205 BX/i18Lx5 probe	General Electric LOGIQ-E9 System, linear 9- to 5-MHz probe	Two-dimensional Aixplorer, SuperLinearTM SL10-2 MHz probe	Acuson S3000 Helx, linear array probe (9L4) or low frequency, curved array probe (4C1)	Siemens S3000 Unit, 9-MHz linear transducer	Acuson S2000, electronic linear array probe (9L4 Transducer) 4–9 MHz	Supersonic Shear Imaging Module, SL15-4 high frequency linear probe
**Muscle**	**SWE m/s (SD)**	**SWE m/s (SD)**	**SWE m/s (SD)**	**SWE m/s (SD)**	**SWE m/s (SD)**	**SWE m/s (SD)**	**SWE m/s (SD)**
**DE**	2.26 (0.49)						
**BB**	1.95 (0.30)	1.76 (0.10)	1.95 (0.22)	2.22 (0.64)			
**ECR**	2.51 (0.37)						
**FDP**	2.30 (0.37)						
**TR**	3.22 (1.19)						
**VA**	1.68 (0.34)	1.76 (0.10)	1.77 (0.15)		4.52 (1.49)		
**BF**	2.31 (0.38)	1.54 (0.12)	1.73 (0.12)				
**TA**	2.56 (0.32)						3.49 (0.58) & 3.86 (0.46)
**GCM**	2.18 (0.43)			1.77 (0.79)			1.89 (0.32) & 2.38 (0.58)
**GCL**				1.77 (0.79)	4.34 (1.56)	1.63 (0.99)	

Here, only Protocol 2 (optimized, rigid SWE) is used for comparison, because the positioning in this protocol was more similar to the other protocols given in this table than Protocol 1 (clinical feasibility). ^ Ewertsen et al. examined both GCM and GCL. The average reported SWV among these two muscle bodies was 1.77 m/s (0.79). * Carpenter et al. used the probe in a transverse orientation, whereas all other studies listed in the table positioned the ultrasound probe longitudinally in relation to the muscle fibers. ^#^ The SWE from Akagi et al. was converted from Pascal to m/s using the equation of *G = ρc_S_*^2^, where *G =* shear modulus (kPa), *ρ* = density of muscle (assumed to be 1.06 kg/m^3^ here). *c_S_* = shear wave speed (m/s) [22]. DE = deltoideus. BB = biceps brachii. ECR = extensor carpi radialis. FDP = flexor digitorum profundus. TR = triceps brachii. VA = vastus lateralis. BF = biceps femoris (caput longum). TA = tibialis anterior. GCM = gastrocnemius (caput mediale).

## Data Availability

The anonymized data and materials are stored locally and any raw data from the statistical analysis can be made available on reasonable request.

## References

[1] Gennisson J.-L., Deffieux T., Fink M., Tanter M. (2013). Ultrasound elastography: Principles and techniques. Diagn. Interv. Imaging.

[2] Bouillard K., Nordez A., Hug F. (2011). Estimation of Individual Muscle Force Using Elastography. PLoS ONE.

[3] Leong H.-T., Ng G.Y., Leung V.Y., Fu S.N. (2013). Quantitative Estimation of Muscle Shear Elastic Modulus of the Upper Trapezius with Supersonic Shear Imaging during Arm Positioning. PLoS ONE.

[4] Cosgrove D.O., Berg W.A., Doré C.J., Skyba D.M., Henry J.-P., Gay J., Cohen-Bacrie C., The BE1 Study Group (2012). Shear wave elastography for breast masses is highly reproducible. Eur. Radiol..

[5] Barr R.G., Nakashima K., Amy D., Cosgrove D., Farrokh A., Schafer F., Bamber J.C., Castera L., Choi B.I., Chou Y.-H. (2015). WFUMB guidelines and recommendations for clinical use of ultrasound elastography: Part 2: Breast. Ultrasound Med. Biol..

[6] Ferraioli G., Filice C., Castera L., Choi B.I., Sporea I., Wilson S.R., Cosgrove D., Dietrich C.F., Amy D., Bamber J.C. (2015). WFUMB guidelines and recommendations for clinical use of ultrasound elastography: Part 3: Liver. Ultrasound Med. Biol..

[7] Lacourpaille L., Hug F., Guével A., Péréon Y., Magot A., Hogrel J.-Y., Nordez A. (2015). Non-invasive assessment of muscle stiffness in patients with duchenne muscular dystrophy: Short Report. Muscle Nerve.

[8] Pichiecchio A., Alessandrino F., Bortolotto C., Cerica A., Rosti C., Raciti M.V., Rossi M., Berardinelli A., Baranello G., Bastianello S. (2018). Muscle ultrasound elastography and MRI in preschool children with Duchenne muscular dystrophy. Neuromuscul. Disord..

[9] Alfuraih A.M., Tan A.L., O’Connor P., Emery P., Wakefield R.J. (2019). The effect of ageing on shear wave elastography muscle stiffness in adults. Aging Clin. Exp. Res..

[10] Bachasson D., Dubois G.J.R., Allenbach Y., Benveniste O., Hogrel J.-Y. (2018). Muscle Shear Wave Elastography in Inclusion Body Myositis: Feasibility, Reliability and Relationships with Muscle Impairments. Ultrasound Med. Biol..

[11] Kronlage C., Grimm A., Romano A., Stahl J.-H., Martin P., Winter N., Marquetand J. (2021). Muscle Ultrasound Shear Wave Elastography as a Non-Invasive Biomarker in Myotonia. Diagnostics.

[12] Gennisson J.-L., Deffieux T., Macé E., Montaldo G., Fink M., Tanter M. (2010). Viscoelastic and Anisotropic Mechanical Properties of in vivo Muscle Tissue Assessed by Supersonic Shear Imaging. Ultrasound Med. Biol..

[13] Alfuraih A.M., O’Connor P., Tan A.L., Hensor E., Emery P., Wakefield R.J. (2017). An investigation into the variability between different shear wave elastography systems in muscle. Med. Ultrason..

[14] Alfuraih A.M., O’Connor P., Hensor E., Tan A.L., Emery P., Wakefield R.J. (2018). The effect of unit, depth, and probe load on the reliability of muscle shear wave elastography: Variables affecting reliability of SWE. J. Clin. Ultrasound.

[15] Bernabei M., Lee S.S.M., Perreault E.J., Sandercock T.G. (2020). Shear wave velocity is sensitive to changes in muscle stiffness that occur independently from changes in force. J. Appl. Physiol..

[16] Crutison J., Klatt D., Sandercock T.G., Perreault E.J., Royston T. (2021). Muscle elastography: Stress versus stiffness. J. Acoust. Soc. Am..

[17] Jenkyn T.R., Ehman R.L., An K.-N. (2003). Noninvasive muscle tension measurement using the novel technique of magnetic resonance elastography (MRE). J. Biomech..

[18] Zonnino A., Smith D.R., Delgorio P.L., Johnson C.L., Sergi F. (2019). MM-MRE: A new technique to quantify individual muscle forces during isometric tasks of the wrist using MR elastography. IEEE Int. Conf. Rehabil. Robot. Proc..

[19] Săftoiu A., Gilja O.H., Sidhu P.S., Dietrich C.F., Cantisani V., Amy D., Bachmann-Nielsen M., Bob F., Bojunga J., Brock M. (2019). The EFSUMB Guidelines and Recommendations for the Clinical Practice of Elastography in Non-Hepatic Applications: Update 2018. Ultraschall Med.-Eur. J. Ultrasound.

[20] Lacourpaille L., Hug F., Bouillard K., Hogrel J.-Y., Nordez A. (2012). Supersonic shear imaging provides a reliable measurement of resting muscle shear elastic modulus. Physiol. Meas..

[21] Dubois G., Kheireddine W., Vergari C., Bonneau D., Thoreux P., Rouch P., Tanter M., Gennisson J.-L., Skalli W. (2015). Reliable Protocol for Shear Wave Elastography of Lower Limb Muscles at Rest and During Passive Stretching. Ultrasound Med. Biol..

[22] Sigrist R.M.S., Liau J., Kaffas A.E., Chammas M.C., Willmann J.K. (2017). Ultrasound Elastography: Review of Techniques and Clinical Applications. Theranostics.

[23] Zaidman C.M., van Alfen N. (2016). Ultrasound in the Assessment of Myopathic Disorders. J. Clin. Neurophysiol..

[24] Alfuraih A.M., O’Connor P., Tan A.L., Hensor E.M.A., Ladas A., Emery P., Wakefield R.J. (2019). Muscle shear wave elastography in idiopathic inflammatory myopathies: A case-control study with MRI correlation. Skeletal Radiol..

[25] Paramalingam S., Needham M., Raymond W., Mastaglia F., Lightowler D., Morin N., Counsel P., Keen H.I. (2021). Muscle shear wave elastography, conventional B mode and power doppler ultrasonography in healthy adults and patients with autoimmune inflammatory myopathies: A pilot cross-sectional study. BMC Musculoskelet. Disord..

[26] Maïsetti O., Hug F., Bouillard K., Nordez A. (2012). Characterization of passive elastic properties of the human medial gastrocnemius muscle belly using supersonic shear imaging. J. Biomech..

[27] Koo T.K., Guo J.-Y., Cohen J.H., Parker K.J. (2013). Relationship between shear elastic modulus and passive muscle force: An ex-vivo study. J. Biomech..

[28] Koo T.K., Guo J.-Y., Cohen J.H., Parker K.J. (2014). Quantifying the passive stretching response of human tibialis anterior muscle using shear wave elastography. Clin. Biomech..

[29] Hug F., Tucker K., Gennisson J.-L., Tanter M., Nordez A. (2015). Elastography for Muscle Biomechanics: Toward the Estimation of Individual Muscle Force. Exerc. Sport Sci. Rev..

[30] Eby S.F., Cloud B.A., Brandenburg J.E., Giambini H., Song P., Chen S., LeBrasseur N.K., An K.-N. (2015). Shear wave elastography of passive skeletal muscle stiffness: Influences of sex and age throughout adulthood. Clin. Biomech..

[31] Šarabon N., Kozinc Ž., Podrekar N. (2019). Using shear-wave elastography in skeletal muscle: A repeatability and reproducibility study on biceps femoris muscle. PLoS ONE.

[32] Gao J., He W., Du L.-J., Chen J., Park D., Wells M., Fowlkes B., O’Dell M. (2018). Quantitative Ultrasound Imaging to Assess the Biceps Brachii Muscle in Chronic Post-Stroke Spasticity: Preliminary Observation. Ultrasound Med. Biol..

[33] Ewertsen C., Carlsen J.F., Christiansen I.R., Jensen J.A., Nielsen M.B. (2016). Evaluation of healthy muscle tissue by strain and shear wave elastography—Dependency on depth and ROI position in relation to underlying bone. Ultrasonics.

